# Anomalous Left Anterior Descending Coronary Artery With Retroaortic Left Circumflex Artery: A Novel Coronary Pattern

**DOI:** 10.1016/j.jaccas.2021.02.012

**Published:** 2021-04-21

**Authors:** Kamel Shibbani, Prashant Nagpal, Ravi Ashwath

**Affiliations:** a Division of Pediatric Cardiology, University of Iowa Stead Family Children's Hospital, Iowa City, Iowa, USA; b Department of Radiology, University of Iowa Hospital and Clinics, Iowa City, Iowa, USA

**Keywords:** congenital heart defect, coronary vessel anomaly, pediatric surgery, ALADCAPA, anomalous left anterior descending coronary artery from pulmonary artery, ALCAPA, anomalous left coronary artery arising from pulmonary artery, CT, computed tomography, FTT, failure to thrive, LAD, left anterior descending, LCA, left coronary artery, LCx, left circumflex, MPA, main pulmonary artery, PA, pulmonary artery, RCA, right coronary artery

## Abstract

A novel coronary anatomy in the form of anomalous left anterior descending coronary artery from pulmonary artery with a retroaortic left circumflex arising from the right coronary artery is presented. This unreported anatomy was discovered in a 7-month-old girl with failure to thrive. (**Level of Difficulty: Intermediate.**)

## Central Illustration

**Figure:**
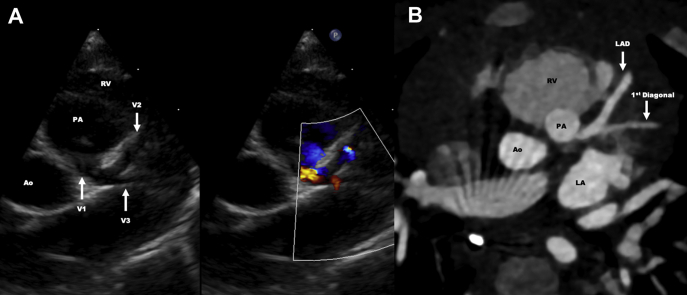

## History of Presentation

A 7-month-old girl presented for a regular checkup and was found to have failure to thrive (FTT), having dropped from the 50th to the 1st percentile for weight. She was admitted for FTT work-up. An incidental murmur on physical exam prompted an electrocardiogram and an echocardiogram. Echo was suspicious for anomalous left coronary artery arising from pulmonary artery (ALCAPA). Physical exam revealed microcephaly, mid-facial hypoplasia, low set ears, periauricular pits, up-slanting palpebral fissures, and a grade II/VI vibratory systolic ejection murmur heard best at the left upper sternal border with no diastolic component or radiation. There was no hepatomegaly. Vitals were as follows: blood pressure: 91/61 mm Hg; pulse: 142 beats/min; temperature: 36.8^o^C; respiration: 40 breaths/min.

**Learning Objectives**

• To create a differential diagnosis of cardiovascular causes of FTT in an infant.
• To create a differential diagnosis for echo findings consistent with ALCAPA.
• To understand the difference in definition and presentation between ALCAPA and ALADCAPA.

## Past Medical History

Our patient was born at 33 weeks’ gestation to a mother with scant prenatal care. Delivery was uncomplicated, but she required a brief admission to the neonatal intensive care unit due to prematurity.

## Differential Diagnoses

The cardiovascular differentials for FTT in the setting of a murmur, especially in a child with minimal perinatal care, must include the following congenital heart disease lesions: ventricular septal defect; atrial septal defect; patent ductus arteriosus; tetralogy of Fallot; and transposition of the great arteries. Of these lesions, tetralogy of Fallot and transposition of the great arteries typically present with some degree of cyanosis. Ventricular septal defect, atrial septal defect, and patent ductus arteriosus can present with clinical signs and symptoms of heart failure, though often they are diagnosed when a murmur is appreciated on exam in the absence of any significant clinical findings. These congenital heart diseases are usually distinguishable by echo.

The echo findings in this case necessitated a vascular differential that included pathologies with abnormal communication to the main pulmonary artery (MPA). Our initial differential therefore included ALCAPA, pulmonary arteriovenous fistula, coronary-cameral fistula with collaterals, and a direct coronary-to-MPA fistula. It is worth noting that while left-to-right shunting is expected in ALCAPA and a coronary-to-MPA fistula, right-to-left shunting is expected in a pulmonary arteriovenous fistula. Other differentials include an internal mammary artery–to–pulmonary artery fistula, as well as other coronary arteriovenous fistulas (1).

## Investigation

**Figure 1:**
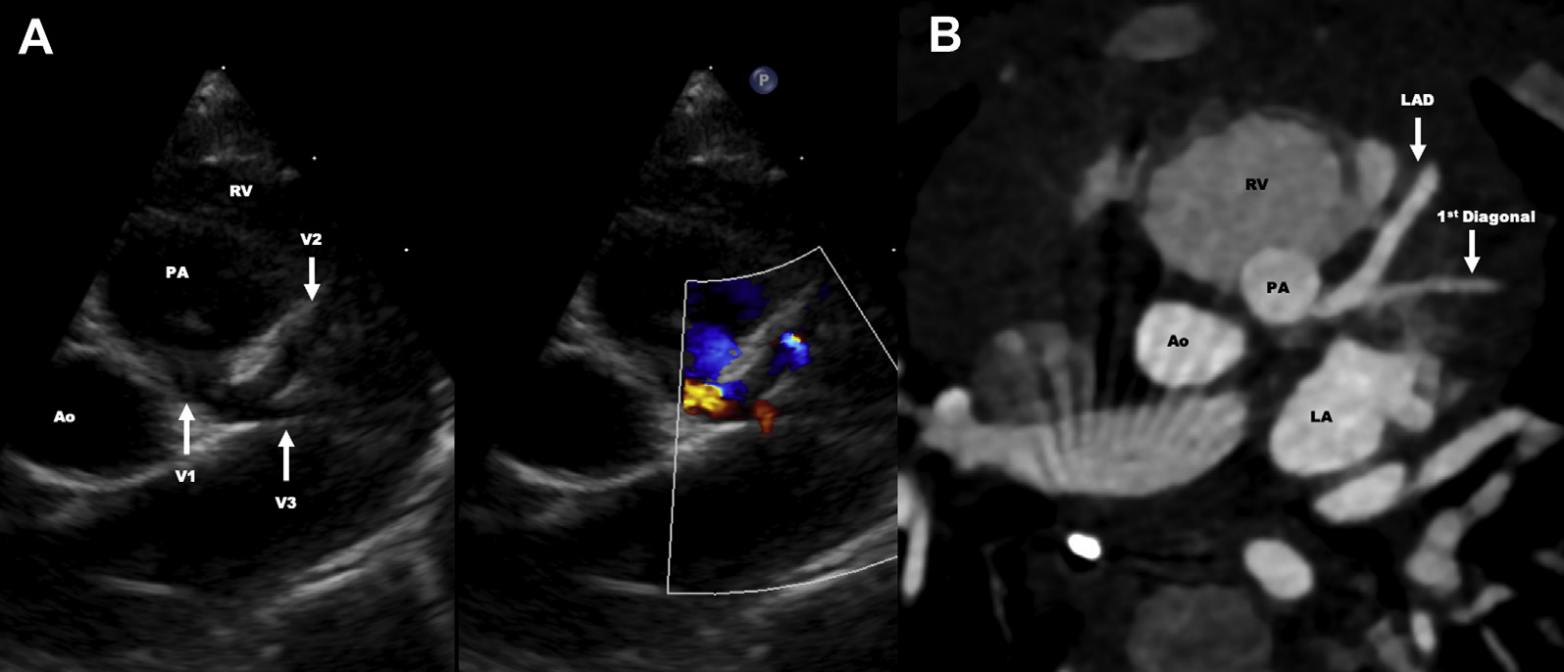
Echocardiography and Computed Tomography Images of the Anomalous Coronary Artery Takeoff From the PA **(A)** Parasternal short-axis view at the level of the base showing a coronary artery taking off from the pulmonary artery (PA) and bifurcating shortly thereafter. On echocardiography, the bifurcation was presumed to be left coronary artery (V1) branching into the left anterior descending (LAD) (V2) and the left circumflex artery (V3). Reversal of flow can be seen at the takeoff of the coronary artery in the Doppler image. **(B)** Axial computed tomographic angiography image confirming the origin of the anomalous vessel from the main PA. However, the vessel originating from the PA, and the bifurcation anatomy was confirmed as LAD coronary artery coursing into the anterior interventricular groove and its first diagonal branch supplying the anterolateral wall of the left ventricle. Ao = aorta; LA = left atrium; RV = right ventricle.

**Figure 2:**
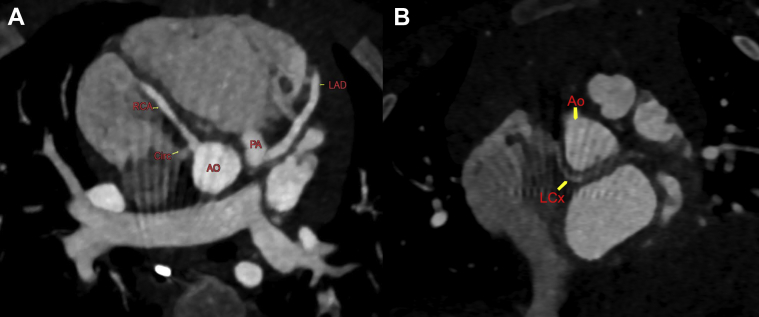
Computed Tomography Detailing Anomalous Coronary Anatomy **(A)** Reformatted oblique axial computed tomography showing all coronary arteries with clear anomalous left anterior descending coronary artery from pulmonary artery anatomy. **(B)** Left circumflex (Circ or LCx) artery is seen originating from the proximal RCA with course posterior to the aorta. Abbreviations as in Figure 1.

An electrocardiogram, echo, genetic testing, complete blood count, chemistry panel, liver function tests, thyroid function tests, and pro–B-type natriuretic peptide were performed. She was found to have mosaic trisomy 21 with unremarkable bloodwork. There were no signs of heart failure or ischemia on electrocardiogram. A repeat transthoracic echo also showed findings suspicious for ALCAPA. A left-sided bifurcating vessel (suspected left coronary artery [LCA] with branching into left anterior descending [LAD] and left circumflex [LCx]) was seen arising from the MPA with reversal of flow noted within the vessel (Figure 1A). To confirm the coronary anomaly and understand the commissural arrangement, a cardiac computed tomography (CT) angiogram was performed. Cardiac CT angiography demonstrated that the echo image showing bifurcation of the LCA system was indeed the LAD and its first diagonal branch (Figure 1B). It demonstrated a unique coronary anatomy: anomalous left anterior descending artery arising from the pulmonary artery (ALADCAPA) with LCx arising from the proximal right coronary artery (RCA) with a retroaortic course (Figures 2A and 2B, Video 1).

## Management

The patient’s anatomy was confirmed intraoperatively. The patient underwent successful transplantation of the LAD to the left aortic sinus. The retroaortic LCx was not surgically corrected.

## Discussion

ALCAPA accounts for <0.5% of all congenital heart diseases. The guidelines set forth by the American Society of Echocardiography define the hallmark findings of ALCAPA on echo as the direct visualization of the LCA arising from the MPA on 2-dimensional echo, retrograde flow from the LCA into the PA on color Doppler, frequently with RCA dilatation (2). Similarly, the diagnostic hallmark of ALCAPA on CT is the direct visualization of the LCA arising from the MPA. Other supporting CT findings include a dilated RCA, dilated intracoronary collaterals, and mitral prolapse (3). The clinical presentation of ALCAPA includes cardiomegaly, tachypnea, congestive heart failure, and mitral regurgitation. Death occurs in up to 85% of uncorrected cases by age 1 year (4). Those that survive into adulthood often are asymptomatic but at risk for sudden death (3).

ALADCAPA is a much rarer variant in which the LAD arises from the MPA and the LCx usually arises from the left aortic sinus. ALADCAPA with a retroaortic LCx arising from the RCA, as reported here, has never been described to the best of our knowledge. In contrast to ALCAPA, patients with ALADCAPA have less severe symptoms due to the LCx providing forward flow to the left ventricular myocardium (5). Though the number of cases is small and a generalization in difficult, patients with ALADCAPA seem to fare better than those with ALCAPA (6). As for the unique anatomy described here, one can assume that there would be minimal differences in the clinical course when compared to the more traditional anatomy of ALADCAPA.

During fetal development, both the aorta and the MPA carry oxygenated blood, so ALCAPA and its variants have no impact on myocardial development. Shortly after birth, the PA pressure and oxygen content drop significantly. Hence, the myocardium that is supplied by the coronary artery arising from the PA (the LCA territory in ALCAPA and the LAD territory in ALADCAPA) will experience hypoxia. To compensate, collateralization from the RCA to the LCA branches ensues (7). Surgical correction of ALCAPA in infants is the management of choice, with direct reimplantation of the LCA to the aorta being the standard of care (7). In our patient, transplantation of the anomalous LAD from MPA to the left aortic sinus was done without any complications. The retroaortic LCx, a benign anomaly, was deemed to be of no immediate or long-term clinical significance and hence was not corrected.

## Follow-Up

The patient had an uncomplicated surgical and post-surgical course and was discharged from the hospital a few days after surgical correction. She was able to gain weight appropriately on fortified formula.

## Conclusions

ALADCAPA is rare and can be associated with other anomalies, such as a retroaortic LCx as seen in this case. Echo is usually the first test in a newborn with suspected coronary anomaly but confirmatory testing with CT may be beneficial to better evaluate the anatomy. Surgery is the treatment of choice.

## Funding Support and Author Disclosures

The authors have reported that they have no relationships relevant to the contents of this paper to disclose.

## Appendix

Supplemental Video 1 Cardiac CT. Axial view showing left anterior descending artery **(yellow arrow)** arising from the main pulmonary artery, with the left circumflex artery **(green arrow)** arising from the right coronary artery with retroaortic course.
